# Case report: Streptomycin combined with dacronin in painless magnifying endoscopy

**DOI:** 10.1097/MD.0000000000035372

**Published:** 2023-10-27

**Authors:** Zheng Zhou

**Affiliations:** a Department of Gastroenterology, The People’s Hospital of Xuancheng City, Anhui Province, China.

**Keywords:** collecting veins, dyclonine, gastric pit, magnifying endoscopy, streptomyces protease

## Abstract

**Rationale:**

To analyze the effect of streptomyces protease combined with dyclonine in painless magnifying endoscopy.

**Patient concerns:**

A total of 100 patients who underwent magnification endoscopy in our hospital from January 2021 to June 2022 were retrospectively analyzed.

**Diagnoses:**

The diagnoses were made by painless magnifying endoscopy and narrow-band imaging combined with pathological findings.

**Interventions:**

The patients were divided into the observation group and control group, the observation group was streptomyces protease combined with dyclonine group, and the control group was dyclonine group, 50 cases in each group. The visibility score under gastroscopy was compared between the 2 groups, and the morphological classification of gastric pit and collecting veins was observed. The detection rates of small lesions and early cancer were compared between the 2 groups. The examination time and adverse reactions were compared between the 2 groups.

**Outcomes:**

Compared with the control group, the streptomyces protease combined with dyclonine group had better clear visibility and a higher detection rate of small lesions, but there was no significant difference in early cancer detection rate between the 2 groups. The examination time of streptomyces protease combined with dyclonine group was relatively prolonged, but there was no significant difference in the incidence of adverse reactions between the 2 groups.

**Lessons:**

Streptomyces protease combined with dyclonine plays a certain role in painless magnifying gastroscopy, which can improve the visibility of gastroscopy, improve the detection rate of small lesions, help to find gastric dysplasia and early gastric cancer diagnosis, and does not increase the incidence of adverse reactions.

## 1. Introduction

This article choose 100 patients who underwent magnification endoscopy from January 2021 to June 2022 were retrospectively analyzed. The patients were divided into streptomyces protease combined with dyclonine group and dyclonine group. In this study, Streptomyces protease combined with dyclonine plays a certain role in painless magnifying gastroscopy, which can improve the visibility of gastroscopy, improve the detection rate of small lesions, help to find gastric dysplasia and early gastric cancer diagnosis, and does not increase the incidence of adverse reactions.

Magnifying gastroscope can better observe the morphology and fine structure of gastric mucosal lesions, and find small gastric lesions, which plays a very important role in early gastric cancer screening.^[1]^The gastric mucosa shows a rich distribution of foamy and mucinous material, which reduces the accuracy of the examination. Therefore, gastroscopy should be cleared of gastric foam and mucus, improve the accuracy of examination. Streptavidin can destroy protein peptide bond and dissolve gastric mucus effectively.^[2]^Dyclonine can eliminate mucus on the surface of gastric mucosa and gas bubbles in gastric cavity.^[3]^ The efficacy and safety of streptavidin combined with Dyclonine mucilage in the treatment of painless magnifying endoscopy were analyzed.

## 2. Materials and methods

### 2.1. General information

A retrospective analysis of 100 patients who underwent magnifying endoscopy from January 2021 to January 2023 was performed. Aged between 30 and 70. There were 52 males,48 females. These patients mainly manifested as epigastric discomfort, heartburn, abdominal distension, abdominal pain, poor appetite symptoms. There were 10 cases complicated with hypertension, 6 cases with diabetes mellitus and 8 cases with cholecystectomy genetic disorder. The patient had taken orally drugs such as acid suppression and stomach protection, and the effect was not good. Some of these patients spoke the local language, and we arranged for someone to finish the communication. The patients were treated with magnifying endoscopy and endoscopy for suspected low-grade and high-grade neoplasia and early cancer. Exclusion criteria: active gastrointestinal bleeding, people with mental disorders, heart, liver, kidney and other important organ function failure, pregnant and lactating woman, and allergic to streptomyces protease and dyclonine mucilage. The patients were divided into 2 groups: the Experimental Group (50 cases) was treated with streptavidin and dyclonine, and the control group (50 cases) was treated with dyclonine.

### 2.2. Treatment

This study was approved by the ethic committee of Xuancheng People Hospital. All patients have signed their informed consent. Patients in both groups fasted for 8 hours before examination, and patients in both groups underwent painless magnifying gastroscopy. Endoscopy for Olympus GIF-290. The patients in the control group were given dyclonine hydrochloride mucilage 10 mL 15 minutes before painless endoscopic therapy, and the patients in the observation group were given dyclonine hydrochloride mucilage 10 mL and streptavidin 20,000 unit orally 2 minutes after oral administration. Most patients felt no obvious discomfort after taking streptavidin and dyclonine hydrochloride mucilage, and a small number of patients had pharyngeal discomfort. Through the telephone and on-site communication to do a good job of patient compliance.

Narrowband imaging combined with painless amplification gastroscopy (NBI ME). After the lesion was found, 0.9% sodium chloride was used to rinse the mucus and foam on the surface of the lesion, and a spray tube was placed through the biopsy hole of the gastroscope. The mucosa was stained with 0.2% indigo carmine and then magnified for observation, microarchitecture and microvasculature were analyzed, and gastric pits were typed and pooled vein morphologies were analyzed, combining the Sakaki classification criteria and the classification method developed by Huang Yonghui.^[4]^ Stomach pits were divided into 5 types, A-type, dot-type pits, B-type, linear pits, c-type, sparse and large linear pits, d-type, plaque pits, e-type, villous pits. The shape of the collecting veins was divided into 3 types^[5]^: R Type (regular type): small veins 0.4 to 0.5 mm in diameter, distributed in a regular spider-like or jellyfish-like pattern; I type (irregular type): vaguely visible collecting veins, irregular shape, type D (vanishing type): collecting veins are not seen under magnifying endoscopy.

### 2.3. Observed indicators

The gastroscopic visibility scores were compared between the 2 groups. The morphological types of gastric pits and collecting veins were observed. The detection rates of small lesion and early cancer were compared between the 2 groups. The examination time was compared between the 2 groups, and the adverse reactions were observed.

### 2.4. Efficacy judgement and detection method

The gastroscopic visibility was scored according to the grading criteria adopted by Kuo et al^[6]^: 1 score: Mucus is very thick, seriously affect the observation field, need more than 50 mL of water to clean. 2 scores: Large amount of mucus adhesion, need <50 mL of water washing. 3 scores: Small amount of mucus adhesion, good visual field. 4 scores: Clear visual field.

The morphological types of gastric pits and collecting veins were observed.

Lesions <5 mm in diameter are called small lesions. The detection of small lesion and early cancer were compared.

The examination time was compared between the 2 groups, and the adverse reactions were observed.

### 2.5. Statistical methods

Statistical Software 22.0 was used. The counting data were tested by chisquare. T test was used for measurement data. *P* < .05 was statistically significant.

## 3. Results

**Figure 1.**
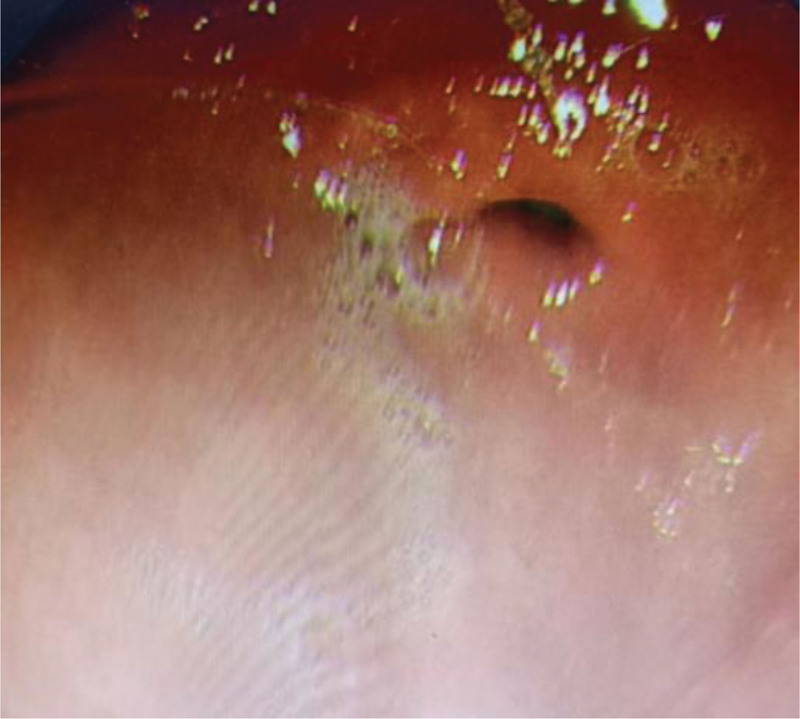
Antrum (control group).

**Figure 2.**
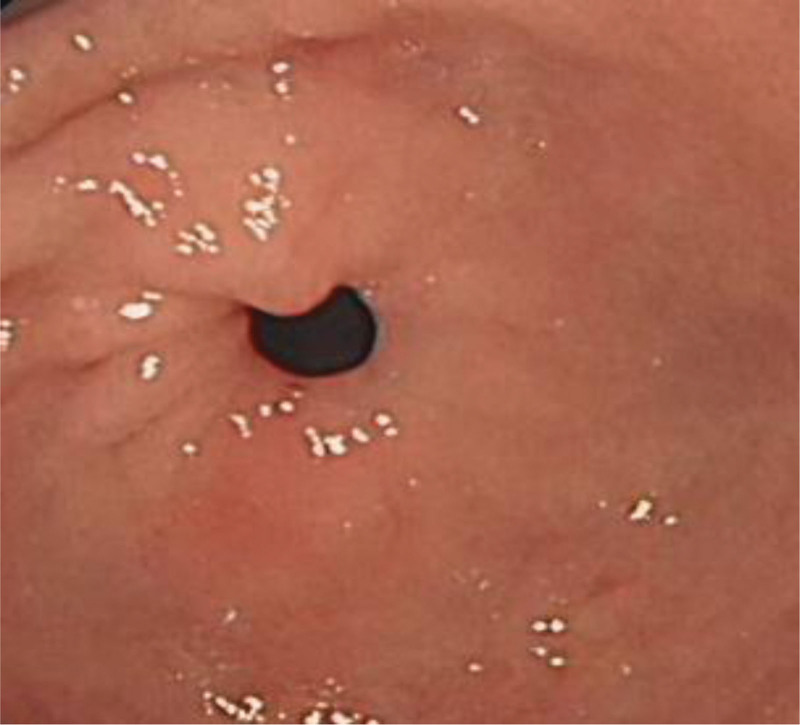
Antrum (observation group).

**Figure 3.**
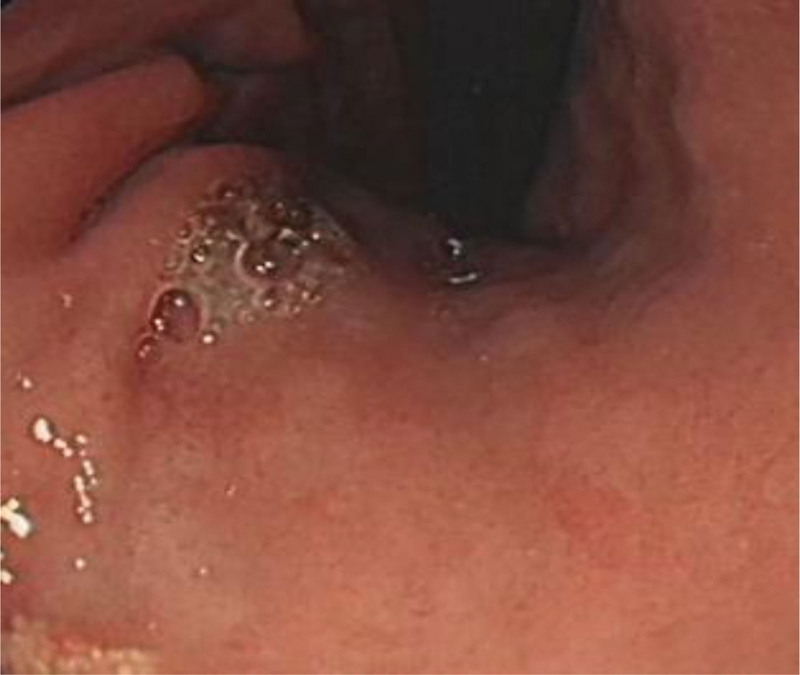
Foundus of stomach (control group).

**Figure 4.**
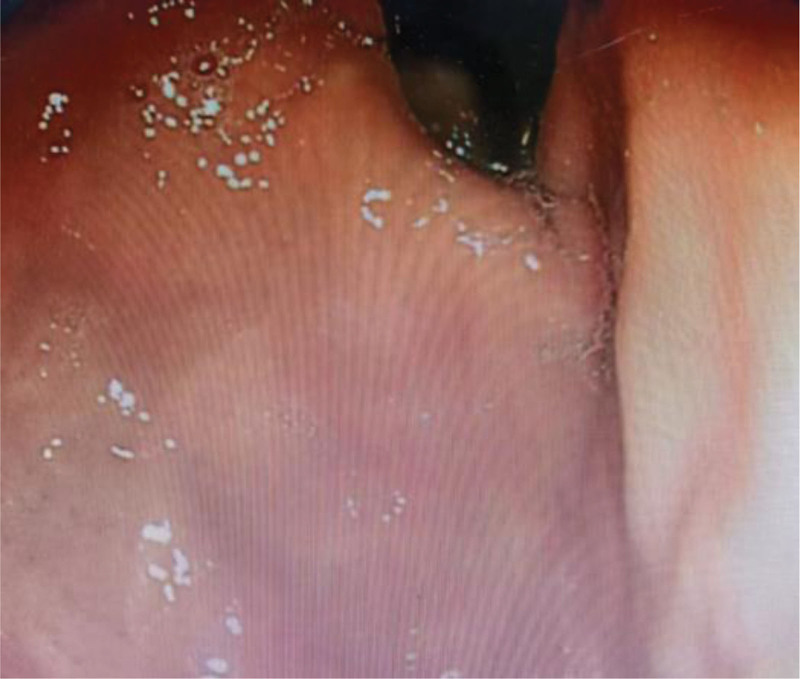
Foundus of stomach (observation group).

**Figure 5.**
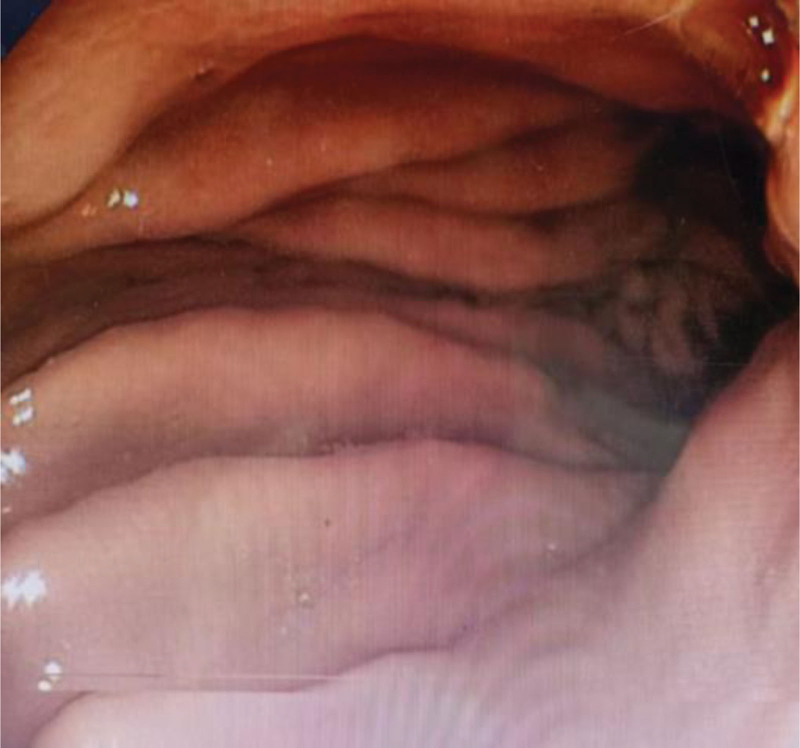
Body of stomach (control group).

**Figure 6.**
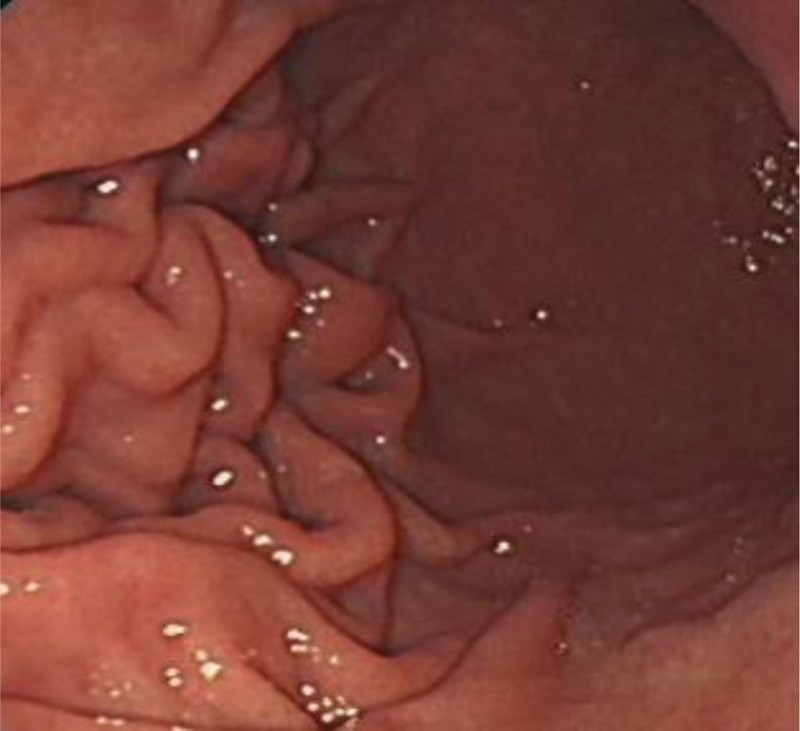
Body of stomach (observation group).

The visual field visibility of gastric lesions in the group of streptavidin and Dyclonine was higher than that in the group of Dyclonine alone. Follows Table 1, Figures 1, 2, 3, 4, 5, and 6.

**Table 1:** The visual field visibility of gastric lesions in the 2 groups.

Group	Number	Foundus of stomach	Body of stomach	Antrum	Total score
Observation group	50	3.30 ± 0.34	3.19 ± 0.28	3.4 ± 0.24	9.814 ± 0.571
Control group	50	2.41 ± 0.21	2.26 ± 0.23	2.40 ± 0.20	7.016 ± 0.356
T value		24.996	28.944	38.137	29.389
*P* value		.000	.017	.092	.000

Morphological classification of gastric pits and collecting veins in 2 groups.

Gastric pits in the Observation Group: type A 17 cases, type B 10 cases, type C 10 cases, type D 8 cases, type E 6 cases.

Gastric pits in the Control Group: type A 20 cases, type B 16 cases, type C 5 cases, type D 4 cases, type E 4 cases.

**Figure 7.**
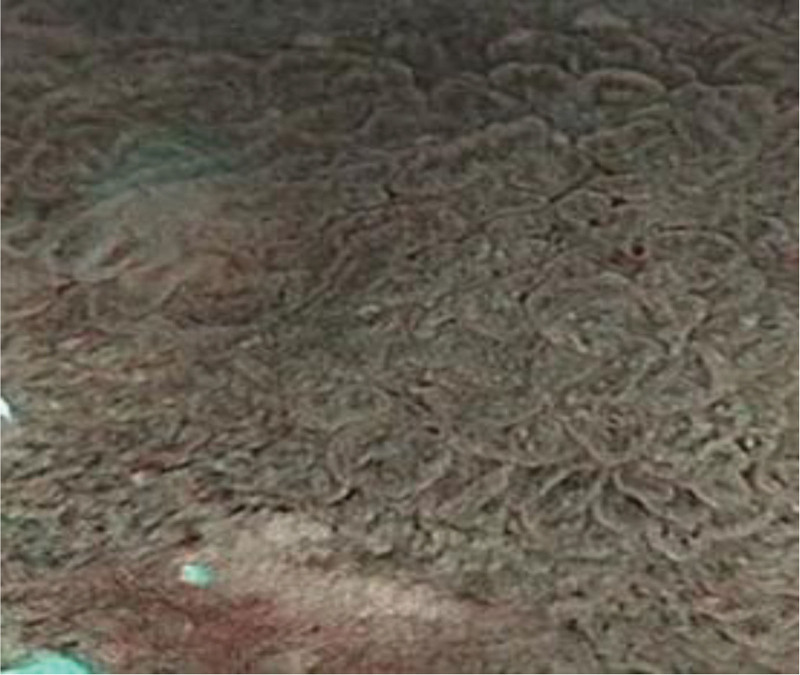
Gastritis pathology.

**Figure 8.**
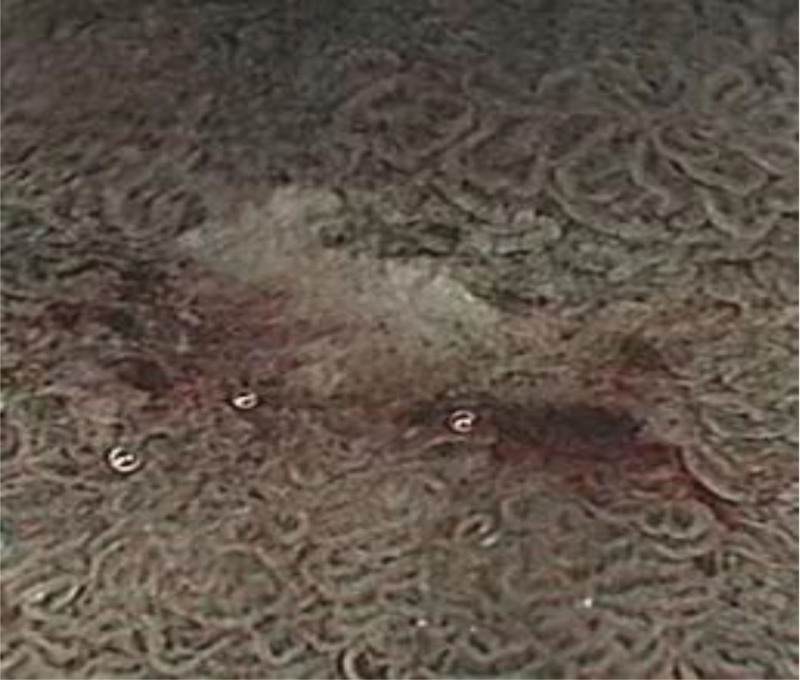
Intestinal metaplasia.

**Figure 9.**
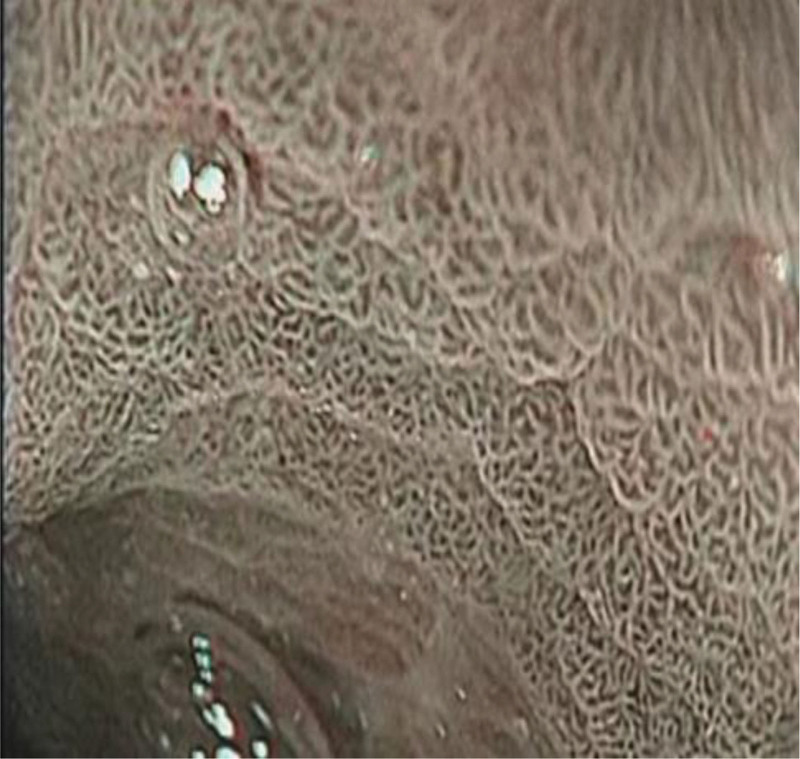
Pathology of early gastric cancer.

**Figure 10.**
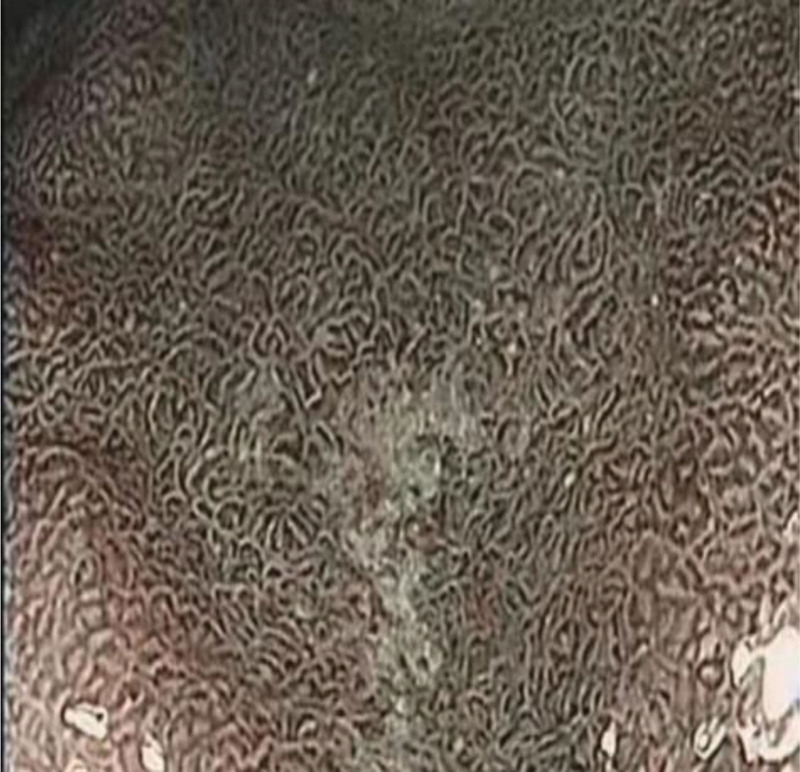
Mild dysplasia.

**Figure 11.**
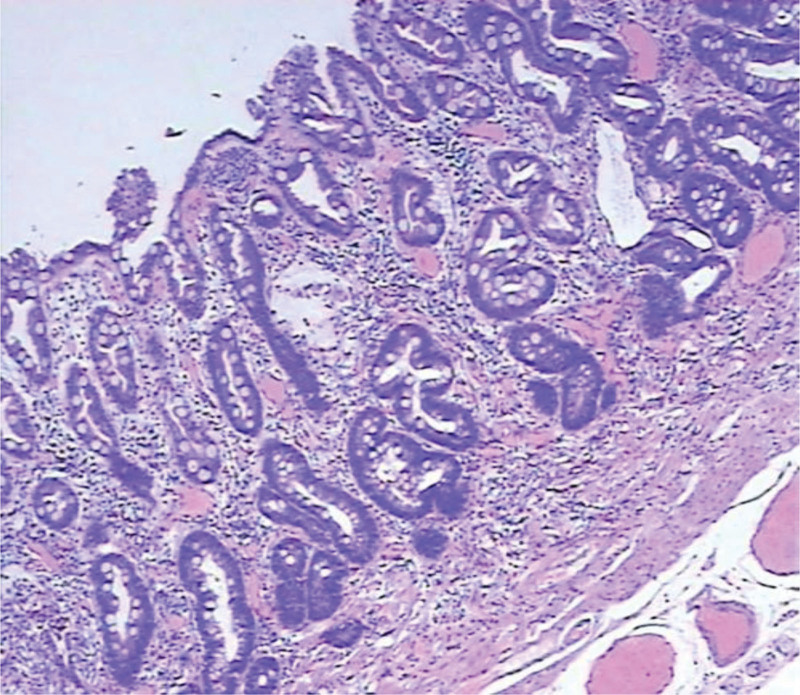
Moderate dysplasia.

**Figure 12.**
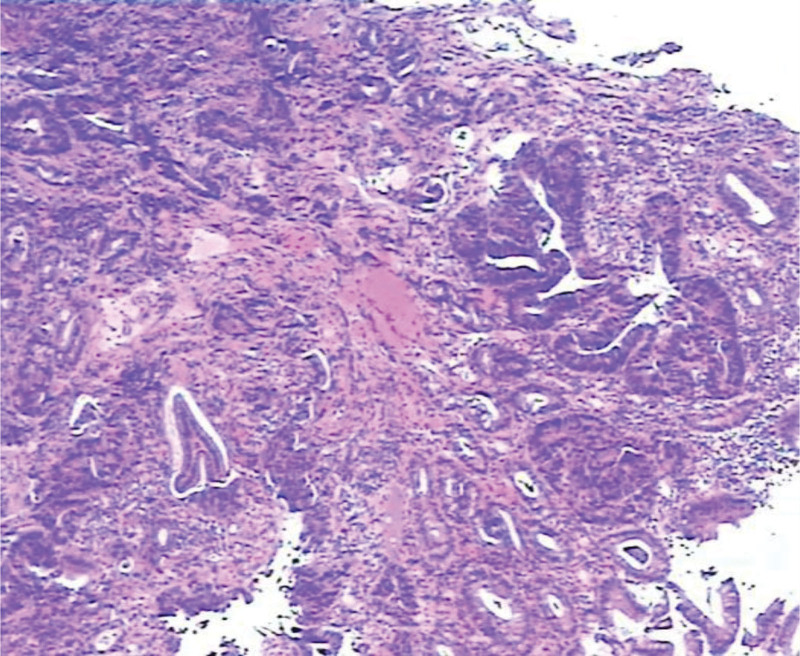
Severe dysplasia.

**Figure 13.**
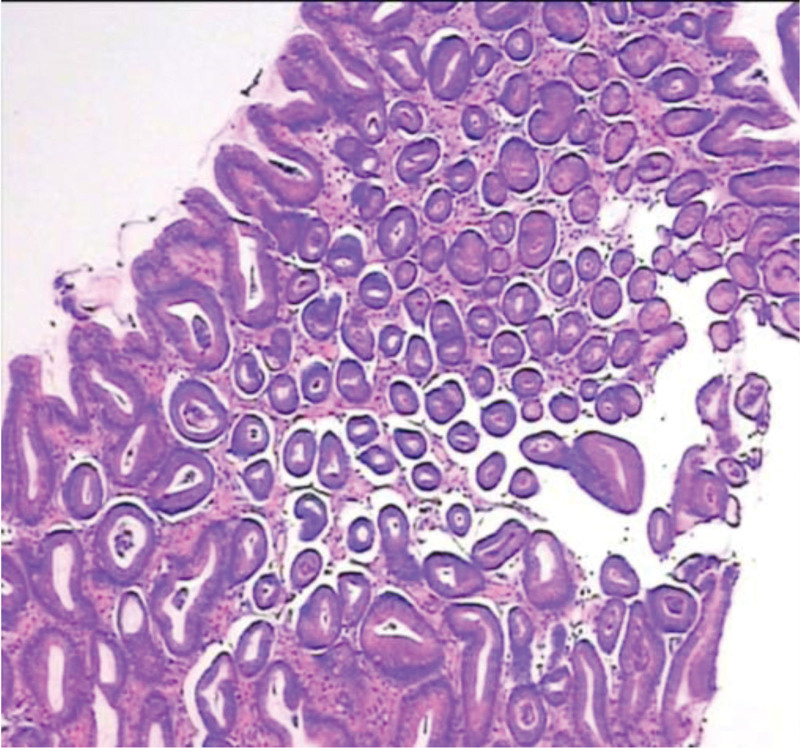
Magnification 100 (control group).

**Figure 14.**
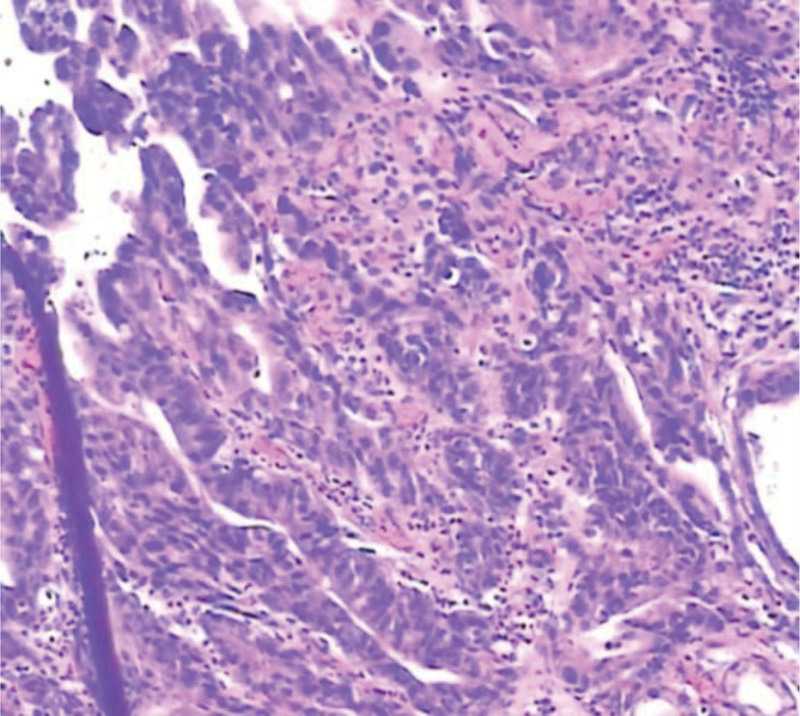
Magnification 100 (observation group).

**Figure 15.**
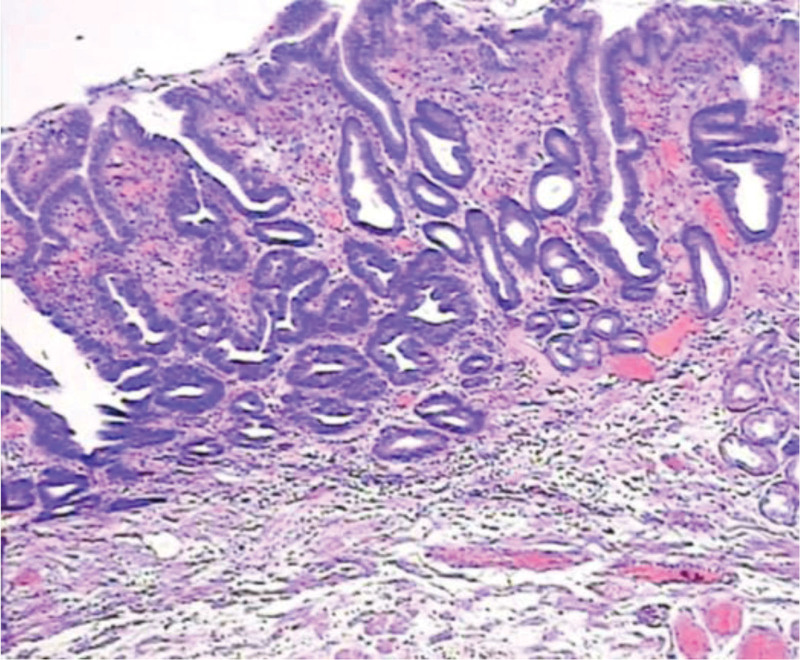
Magnification 80 (control group).

**Figure 16.**
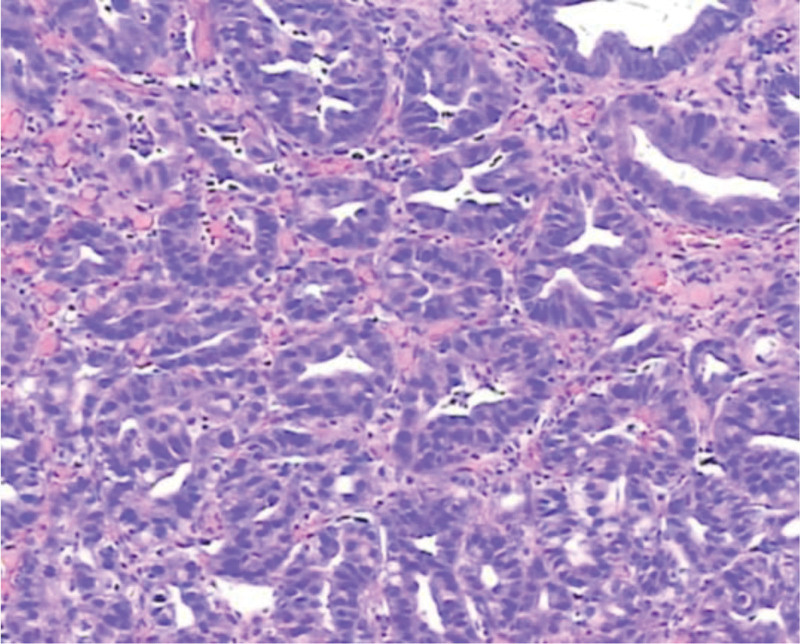
Magnification 80 (observation group).

Studies have shown that Gastric pit type is associated with histopathology. In this study, 37 cases of A-type gastric pits were found in normal gastric mucosa and inflammatory changes. The inflammatory changes can be seen in Figure 7. Type B gastric pits in 26 cases, 2 cases of intestinal metaplasia, no dysplasia. Intestinal metaplasia was found in all 15 cases of type C gastric pits, including 5 cases of dysplasia. This intestinal metaplasia can be seen in Figure 8. There were 12 cases of type D gastric pits, intestinal metaplasia and 6 cases of dysplasia. There were 10 cases of E-type gastric pits with intestinal metaplasia, 2 cases of moderate dysplasia, 2 cases of severe dysplasia, 6 cases of early gastric cancer. The histopathology of early gastric cancer can be seen in Figure 9. Type C gastric pits may indicate mild dysplasia, which can be seen in Figure 10. Type D gastric pits may indicate moderate dysplasia and can be seen in Figure 11. The E-type gastric pits may indicate severe dysplasia, which can be seen in Figure 12. The percentage of C-D gastric pits was 48% in the observation group and 26% in the control group. The chi-square test by SPSS software showed that the chi-square value was 5.191, *P* value was .023, the difference was statistically significant, it is indicated that the detection rate of dysplasia and early gastric cancer is increased in the observation group. Follows Table 2, Figures 13, 14, 15, and 16.

**Table 2:** The relationship between gastric pit type and histopathology.

Gastric pit type	Number	Normal/chronic inflammatory	Intestinal metaplasia	Mild dysplasia	Moderate dysplasia	Severe dysplasia	Early gastric cancer
A	37	37					
B	26	24	2				
C	15		10	3	2		
D	12		6	2	2	2	
E	10				2	2	6
Total	100	61	18	5	6	4	6

Morphological classification of collecting vein in observation group:

R type 18 cases, I type 20 cases, D type 12 cases.

Morphological classification of collecting vein in control group:

R type 23 cases, I type 19 cases, D type 8 cases.

The study showed that the shape of collecting veins was related to Helicobacter pylori infection. The shape of collecting veins of type R suggested normal gastric mucosa, while that of type I suggested partial Helicobacter pylori infection, pattern D of collecting veins suggests Helicobacter pylori infection. In this study, 41 cases were Type R, 38 cases were HP negative, 3 cases were HP positive, 39 cases were type I, 31 cases were HP negative, 8 cases were HP positive, 20 cases were Type D, 18 cases were HP positive, hP was negative in 2 cases. There were 32 cases (64%) of type D + Type I in Observation Group and 27 cases (54%) of type D + Type I in control group, there was no significant difference in the Helicobacter pylori infection rate between the control group and the observation Group. Follows Table 3.

**Table 3:** The relationship between the shape of collecting vein and HP infection.

Shape of collecting vein	HP positive number	HP negative number	Total
R	3	38	41
I	8	31	39
D	18	2	20
Total	29	71	100

The detection rate of small lesions in streptavidin plus dyclonine group was significantly higher than that in control group (χ^2^ = 4.026, *P* = .045), but there was no significant difference in the detection rate of early cancer between the 2 groups. Follows Table 4.

**Table 4:** The detection of small lesion and early cancer in 2 groups.

	Number	Small lesion number	Early cancer number
Observation group	50	32	4
Control group	50	22	2
χ^2^		4.026	0.709
*P*		.045	.400

The time of examination in streptavidin plus dyclonine group was longer than that in control group (t = 3.247, *P* = .002). The difference was statistically significant, but there was no significant difference in the incidence of adverse reactions between the 2 groups. Follows Table 5.

**Table 5:** To compare the examination time of the 2 groups and observe the adverse reactions.

	Examination time	Allergy	Shock	Infection	Total of adverse recations
Observation group	6.150 ± 0.286	1	1	5	7
Control group	5.988 ± 0.207	1	1	4	6
Statistics	t = 3.247				χ^2^ = 0.088
*P*	.002				.766

## 4. Conclusion

Early gastric cancer is limited to the mucosa or submucosa, early gastric cancer can be treated by endoscopic mucosal resection or endoscopic submucosal dissection, the prognosis is good, quality of life is high. Magnifying gastroscopy (ME) can observe the micro-structural changes of gastric mucosa, such as opening of glandular ducts and microvessels, analyze the micro-structural changes of gastric mucosa, and make early diagnosis of early gastric cancer and precancerous lesions.^[7,8]^

Narrow-band imaging (NBI) is a technique that uses a filter to filter out the broad-band spectrum of the red, blue and green waves emitted by an endoscopic light source, leaving only the narrow-band spectrum for diagnosis of various diseases of the digestive tract, not only can accurately observe the morphology of digestive tract mucosa epithelium, such as the structure of epithelial glandular pits, but also can observe the morphology of epithelial vascular network.^[9,10]^ Magnifying gastroscopy requires a clear field of vision, which is mainly affected by the mucus and foams on the gastric mucosa, the main components of which are glycoproteins. Streptavidin can destroy the protein peptide bond, dissolve the gastric mucus effectively, and improve the clarity of gastroscopy. Studies have shown that streptavidin can reduce the amount of mucus in the gastric cavity, improve the visibility of gastroscopy and the detection rate of small lesions.^[11,12]^ Dyclonine hydrochloride is a Topical anesthetic that can enhance the anesthetic effect of General anesthetic at the site of gastroscopy, inhibit the upper gastrointestinal reflex, and reduce mucus secretion, improve the clarity of visual field inspection.^[3,13]^ Magnifying gastroscope magnification of about 60 to 170 times, can show the surface structure of gastric mucosa, such as pit opening of glandular duct, mucosal microvascular morphology, such as the shape of collecting veins. Studies have shown that gastric pits type A and type B represent pits in normal and mild inflammation, with type a mostly located in the body and fundus of the stomach and type B mostly located in the pylorus of the antrum; Type C and D were formed by spreading and connecting of gland orifices in gastric mucosal lesions such as inflammation, edema, erosion and ulceration, and

had intestinal metaplasia and mild-moderate dysplasia, severe dysplasia was more common in Type D and E, and early gastric carcinoma was type E.^[14,15]^ Studies have shown that normal gastric body mucosa without Helicobacter pylori infection exhibits a regular distribution of collecting veins (Type R), the symptoms of Helicobacter pylori gastritis are absence of collecting veins (type d) or irregular collecting veins (type I).^[16,17]^ Other studies have shown that the infection rate of Helicobacter pylori in collecting veins of the regular type of gastric body mucosa is 5.6%, and that of Helicobacter pylori in collecting veins of the irregular type is 71.9%, the Helicobacter pylori infection rate was 82.4%.^[18]^ In this paper, the visual field visibility of gastric lesions in the group of streptavidin plus Dyclonine was higher than that in the group of Dyclonine alone. The visual field clarity was better and the detection rate of small lesions was higher in the group of streptavidin plus dyclonine, the difference was statistically significant. The microstructures of gastric mucosa were observed by magnifying gastroscope. In the group of streptavidin and dyclonine, the percentage of C-D gastric pits was 48%, while in the control group, the percentage of C-D gastric pits was 26%. The chi-square test showed that the chi-square value was 5.191, *P* = .023; The difference was statistically significant, indicating that the detection rate of dysplasia and early gastric cancer was increased in the observation group. The shape of collecting vein in gastric mucosa was observed. In the group of streptavidin plus dyclonine, there were 32 cases (64%) of type D + Type I collecting vein, the chi-square test showed that the chi-square value was 1.033, *P* = .309. There was no significant difference in the Helicobacter pylori infection rate between the 2 groups. The above study showed that the streptavidin plus dyclonine group had better clear visibility and higher detection rate of small lesions, but there was no significant difference in the detection rate of early cancer between the 2 groups. The examination time of streptavidin combined with Dyclonine group was longer, but there was no significant difference in the incidence of adverse reactions between the 2 groups. Through the above research, streptavidin and dyclonine play a certain role in painless magnifying gastroscopy, which can improve the clear visibility of the field of vision, and increase the detection rate of small lesions, it is helpful for the diagnosis of gastric dysplasia and early gastric cancer without increasing the incidence of adverse reactions.

## Author contributions

**Writing – original draft:** Zheng Zhou.

**Writing – review & editing:** Zheng Zhou.
